# Tetraspecific scFv construct provides NK cell mediated ADCC and self-sustaining stimuli via insertion of IL-15 as a cross-linker

**DOI:** 10.18632/oncotarget.12073

**Published:** 2016-09-16

**Authors:** Joerg U. Schmohl, Martin Felices, Deborah Todhunter, Elizabeth Taras, Jeffrey S. Miller, Daniel A. Vallera

**Affiliations:** ^1^ University of Minnesota, Masonic Cancer Center, Section of Molecular Cancer Therapeutics, Therapeutic Radiology-Radiation Oncology, University of Minnesota, Minneapolis, MN, USA; ^2^ University of Tuebingen, Department for Hematology and Oncology, Medicine Department 2, University Hospital of Tuebingen, Tuebingen, Germany; ^3^ University of Minnesota, Department of Medicine, Division of Hematology, Oncology, and Transplantation, Minneapolis, MN, USA

**Keywords:** TetraKE, EpCAM, CD133, carcinoma, cancer stem cells

## Abstract

**Background:**

The design of a highly effective anti-cancer immune-engager would include targeting of highly drug refractory cancer stem cells (CSC). The design would promote effective antibody-dependent cell-mediated cytotoxicity (ADCC) and simultaneously promote costimulation to expand and self-sustain the effector NK cell population. Based on our bispecific NK cell engager platform we constructed a tetraspecific killer engager (TetraKE) comprising single-chain variable fragments (scFvs) binding FcγRIII (CD16) on NK cells, EpCAM on carcinoma cells and CD133 on cancer stem cells in order to promote ADCC. Furthermore, an Interleukin (IL)-15-crosslinker enhanced NK cell related proliferation resulting in a highly active drug termed 1615EpCAM133.

**Results:**

Proliferation assays showed TetraKE promoted proliferation and enhanced NK cell survival. Drug-target binding, NK cell related degranulation, and IFN-γ production was specific for both tumor related antigens in EpCAM and CD133 bearing cancer cell lines. The TetraKE showed higher killing activity and superior dose dependent degranulation. Cytokine profiling showed a moderately enhanced IFN-γ production, enhanced GM-CSF production, but no evidence of induction of excessive cytokine release.

**Methods:**

Assembly and synthesis of hybrid genes encoding the TetraKE were performed using DNA shuffling and ligation. The TetraKE was tested for efficacy, specificity, proliferation, survival, and cytokine production using carcinoma cell lines and functional assays measuring NK cell activity.

**Conclusion:**

1615EpCAM133 combines improved induction of ADCC with enhanced proliferation, limited cytokine response, and prolonged survival and proliferation of NK cells. By linking scFv-related targeting of carcinoma and CSCs with a sustaining IL-15 signal, our new construct shows great promise to target cancer and CSCs.

## INTRODUCTION

Immune engagers show promising results in cancer treatment and already demonstrate some clinical success. T cells as well as NK cells are the main effector cells that can be harnessed for cancer defence. Evidence now supports that Bispecific T cell engagers (BiTEs) are highly effective in targeting CEA with MT111 for colorectal, ovary, and stomach cancer [1], CD19 for acute lymphatic leukemia (Blinatumomab) [2] and EpCAM for cancer related ascites (Catumaxomab). However BiTEs can induce harmful cytokine release related toxicity leading to patient disorders, thereby limiting the dose [3]. In BiTEs, CD3 engagement induces interleukin (IL)-2 release contributing to cytokine toxicity. We believe that engaging NK effector cells may provide a better safety profile *in vitro* and *in vivo*. In order to engage NK cells, bispecific NK cell engagers (BiKEs) were synthesized forming an immune synapse between NK cells and cancer cells [4–8]. A problem is that all of these BiKEs are limited by an inability to facilitate NK cell expansion, as CD16 ligation does not trigger proliferation. To resolve this issue we incorporated modified IL-15 as a cross-linker between the scFv to form an IL-15 TetraKE that could induce expansion and proliferation. IL-15 is arguably the best-known natural regulator of NK cell homeostasis and development and is therefore an important factor in NK-mediated immune surveillance [9].

NK cells are cytotoxic lymphocytes widely acknowledged for their potential to control cancer and for their role in immunosurveillance [10]. NK cells are regulated by a repertoire of inhibitory and activation surface receptors. They have the ability to recognize stressed cells in the absence of MHC by stimulation of activating receptors such as NKG2D and natural cytotoxicity receptors. NK cells also highly express CD16, a potent activation receptor that binds to the Fc portion of IgG antibodies, which has a central role in providing antibody-dependent cell-mediated cytotoxicity (ADCC) [11]. NK cells are regulated by IL-15, which can induce increased antigen dependent cytotoxicity, lymphokine-activated killer activity and also mediation of Interferon (IFN), tumor-necrosis factor (TNF) and Granulocyte-macrophage colony-stimulating factor (GM-CSF) responses [12–14]. All of these IL-15 activated functions contribute to improved cancer defense (as reviewed in [15]).

In this study, we targeted the epithelial cell adhesion molecule (EpCAM) with an anti-EpCAM scFv and CD133 with anti-CD133 scFv. The EpCAM molecule is known to be a marker of great therapeutic interest for targeting carcinoma and is overexpressed in a myriad of different carcinoma tissues such as colon, ovary, breast and prostate cancer. Involvement in prognostically relevant factors like enhanced tumor proliferation, resistance to chemo- and radiotherapy, reduced overall-survival, and selective expression on epithelial cells, make EpCAM a valuable marker for cancer targeting [16, 17]. EpCAM is connected to the Wnt/β-catenin pathways, implying relevance in physiologic and cancer stem-cell (CSC) regulation.

CD133 was targeted because it is expressed on CSC that cause drug resistant relapse by promoting tumor initiation and self-propagation of cancer cells. CD133 is a pentaspan transmembrane protein which is also associated with the Wnt/ß-catenin pathway and thus with cell proliferation [18, 19]. CD133 has a negative impact in patient survival when detected on cancer cells [20, 21]. Drugs have been created targeting CD133 including targeted toxins (^C178A^BC-CD133Mab, dEpCAMCD133KDEL, CD133NPs, dCD133KDEL) [22–25] and BiKEs (CD16133) [4] showing high efficacy in tumor elimination *in vitro* and *in vivo* even in tumors with only a small number of CD133-expressing cells.

In this paper, we engineered the TetraKE 1615EpCAM133 to simultaneously engage EpCAM and CD133 increasing its targeting capability to target cancer cells and CSC alike. By including IL-15 in our construct we improved the action by rendering the molecule capable of NK cell expansion. We show that our TetraKE is highly specific against EpCAM and CD133 bearing cells, is capable of both NK cell mediated ADCC and NK cell expansion, and represents a promising new therapeutic modality.

## RESULTS

### Engineering of 1615EpCAM133

**Figure 1:**
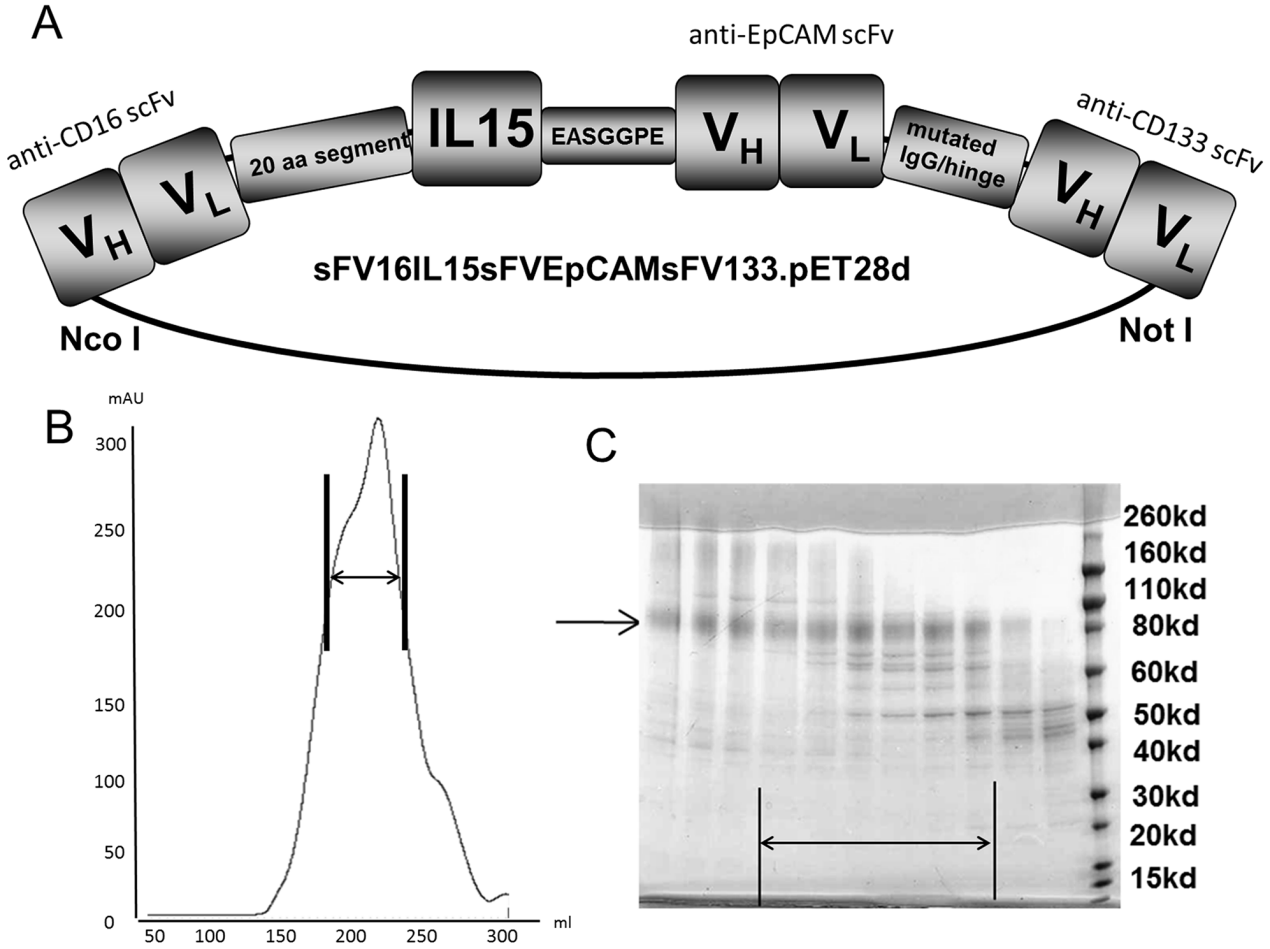
Construction and purification **A.** Construction of tetraspecific hybrid protein 1615EpCAM133 NK cell engager (TetraKE). From left to right, the plasmid contains V_H_ and V_L_ regions of anti-CD16 spliced to a 20 amino acid (aa) PSGQAGAAASESLFSNHAY linker, then IL-15, EASGGPE, the V_H_ and V_L_ region of anti-EpCAM, mutated IgG/hinge linker, and then the V_H_ and V_L_ of anti-CD133 to form 1615EpCAM133 TetraKE. **B.** Size exclusion data from the fast flow sepharose procedure (arrow marks appropriate drug size range). **C.** SDS-PAGE of isolated protein (marked with arrow).

The design of the engineered protein is shown in Figure 1A. After harvesting and refolding processes fast flow sepharose procedure was performed and showed an appropriate size related peak as shown in Figure 1B. Densitometric evaluation of purity revealed 90% when analyzed in SDS-page. Compared to the molecular weight (MW) standard the produced protein showed a MW of 95,900 Da and confirmed the sequence derived size estimation (Figure 1C).

### Specificity in binding

**Figure 2:**
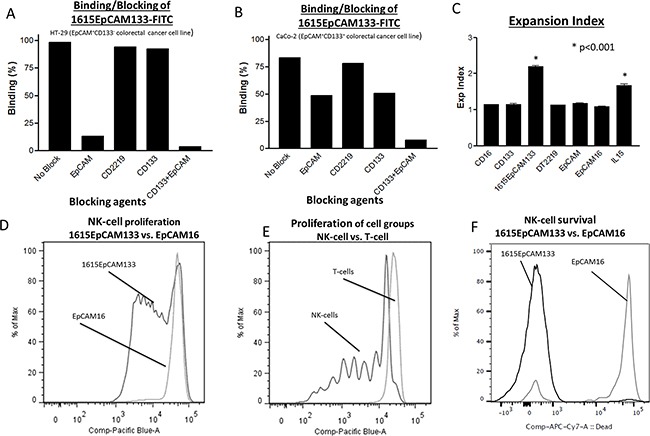
Binding specificity, biologic validation of IL-15 moiety **A.** Binding assays against HT-29 cells and **B.** Caco-2 cells were performed using FITC-labeled 1615EpCAM133 TetraKE (200nM) competed with excess unlabeled noted scFvs (1000nM respectively). Experiments were repeated with 500nM of 1615EpCAM133. Results were reproducible. **C.** Purified NK cells were stained with Celltrace and cocultured with an anti-CD16 scFv [CD16], anti-CD133 scFv [CD133], 1615EpCAM133 TetraKE, DT2219 (mutated diphtheria toxin linked to an anti-CD22 and an anti-CD19 scFv), anti-EpCAM scFv [EpCAM], EpCAM16 BiKE or IL-15 [IL15] for 7 days (n=5). Graph shows pooled data of the expansion index for each of the groups. **D.** Representative histogram of PBMCs stained with Celltrace dye and cocultured with 50 nM of 1615EpCAM133 TetraKE or EpCAM16 BiKE for 7 days. **E.** Representative histogram comparing proliferation of CD56^+^CD3^−^ NK cells with CD56^−^CD3^+^T cells. **F.** Representative histogram illustrating survival (by means of Live/Dead dye exclusion) of purified NK cells exposed to the 1615EpCAM133 TetraKE or EpCAM16 BiKE for 7 days. Dead cells display inclusion of the dye (high peak) while live cells exclude it (low peak). P-values were estimated with one-way-ANOVA values are presented with standard deviation.

To evaluate specificity of our drug, flow cytometry based blocking assays were performed with HT-29 (EpCAM^+^, CD133^−^) and Caco-2 (EpCAM^+^, CD133^+^) colon carcinoma cell lines. In this assay a FITC-labeled 1615EpCAM133 TetraKE competes with saturating concentrations of unlabeled anti-EpCAM scFv, DT2219, anti-CD133 scFv, and a combination of anti-CD133 scFv and anti-EpCAM scFv (1000nM respectively). In HT-29 cells, the TetraKE bound >98% of cells. Blocking with either anti-EpCAM scFv or anti-EpCAM combined with anti-CD133 scFv led to a reduction in binding, whereas anti-CD133 scFv alone as well as the control drug DT2219 showed minimal blocking capability (HT-29 cells do not express CD22 and CD19 and a minimum of CD133) (Figure 2A). In Caco-2 cells, where TetraKE binds >83%, blocking with either anti-EpCAM or anti-CD133 scFv led to a reduction of binding. Blocking with anti-EpCAM combined with anti-CD133 scFv led to the highest level of blocking since both tumor related antigens are targeted by the TetraKE (Figure 2B). The control with CD2219 (a bispecific antibody consisting of anti-CD22 scFv spliced to anti-CD19 scFv) showed no blocking capability. Experiments were repeated with 500nM of 1615EpCAM133. Results were reproducible.

#### Biologic validation of IL-15 moiety

The capability of the IL-15 moiety within the drug to induce proliferation and survival is shown in Figure 2C-2F. We exposed purified NK cells to 50 nM of an anti-CD16 scFv, anti-CD133 scFv, 1615EpCAM133 TetraKE, DT2219 (mutated diphtheria toxin linked to an anti-CD22 and an anti-CD19 scFv), anti-EpCAM scFv, EpCAM16 BiKE and IL-15 (NCI). Expansion index, which determines overall expansion of the culture, showed significantly enhanced expansion in the TetraKE and in the IL-15 groups (p<0.001), (Figure 2C). To compare the ability of our modified IL-15 linker to induce proliferation, PBMCs or purified NK cells were cultured after staining with a reactive dye and exposed to 50 nM of EpCAM16 BiKE or 1615EpCAM133 TetraKE. After incubation, flow cytometry analysis was performed on gated CD56^+^ CD3^−^ cells to evaluate NK cells and on CD56^−^CD3^+^ cells to evaluate T-cells. In Figure 2D, only NK cells treated with the TetraKE showed substantial proliferation. Treatment with EpCAM16 BiKE did not. The ability to induce specific proliferation to NK cells is shown in Figure 2E. T-cells did not proliferate after exposure to TetraKE. To study the ability of the TetraKE to enhance survival of NK cells, purified NK cells were cultured for 7 days with 30 nM of 1615EpCAM133 TetraKE or EpCAM16 BiKE. At the end of culture viability staining, using Live/Dead Dye, showed much higher percentages of live NK cells in the TetraKE group (Figure 2F).

### Activity of 1615EpCAM133

**Figure 3:**
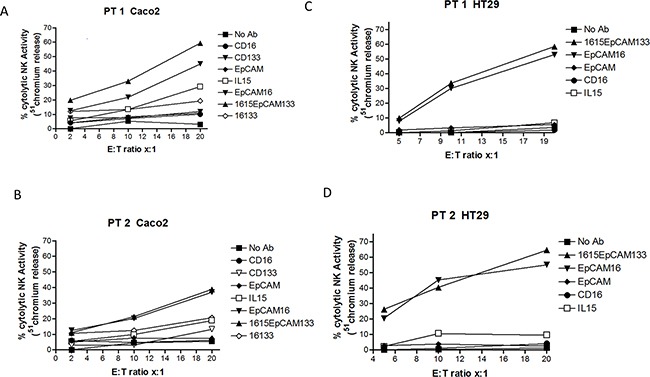
Activity of 1615EpCAM133 TetraKE **A, B.** The activity of the 1615EpCAM133 TetraKE was evaluated with ^51^chromium release assays. Freshly isolated NK cells from two donors were added to the human colorectal carcinoma cell line Caco-2 (CD133^+^, EpCAM^+^). Cells were co-cultured with targets at noted effector to target (E:T) ratios for 4 hours and ^51^chromium release was then evaluated. In **C** and **D.** NK cells from two donors were exposed to human colorectal carcinoma cell line HT-29 (EpCAM^+^, CD133^−^) and ^51^chromium release was measured in the same manner as described above.

1615EpCAM133 activity was evaluated with standard ^51^chromium release assays in order to measure the drug's ability to mediate NK cell killing of cancer cells. Therefore we used Caco-2 (CD133^+^, EpCAM^+^) and HT-29 (CD133^−^, EpCAM^+^) targets and performed the assay with freshly isolated NK cells of two donors for each cancer cell line (Figure 3A, 3B) and (3C, 3D) respectively. Beside 1615EpCAM133 TetraKE, no antibody, anti-CD16 scFv, anti-CD133 scFv, anti-EpCAM scFv, IL-15 alone and EpCAM16 BiKE were added as controls. In all donors and in both cancer cell types, 1615EpCAM133 and EpCAM16 showed superior killing when compared to other controls at increasing Effector:Target (E:T) ratios.

### Dose dependent degranulation capability

**Figure 4:**
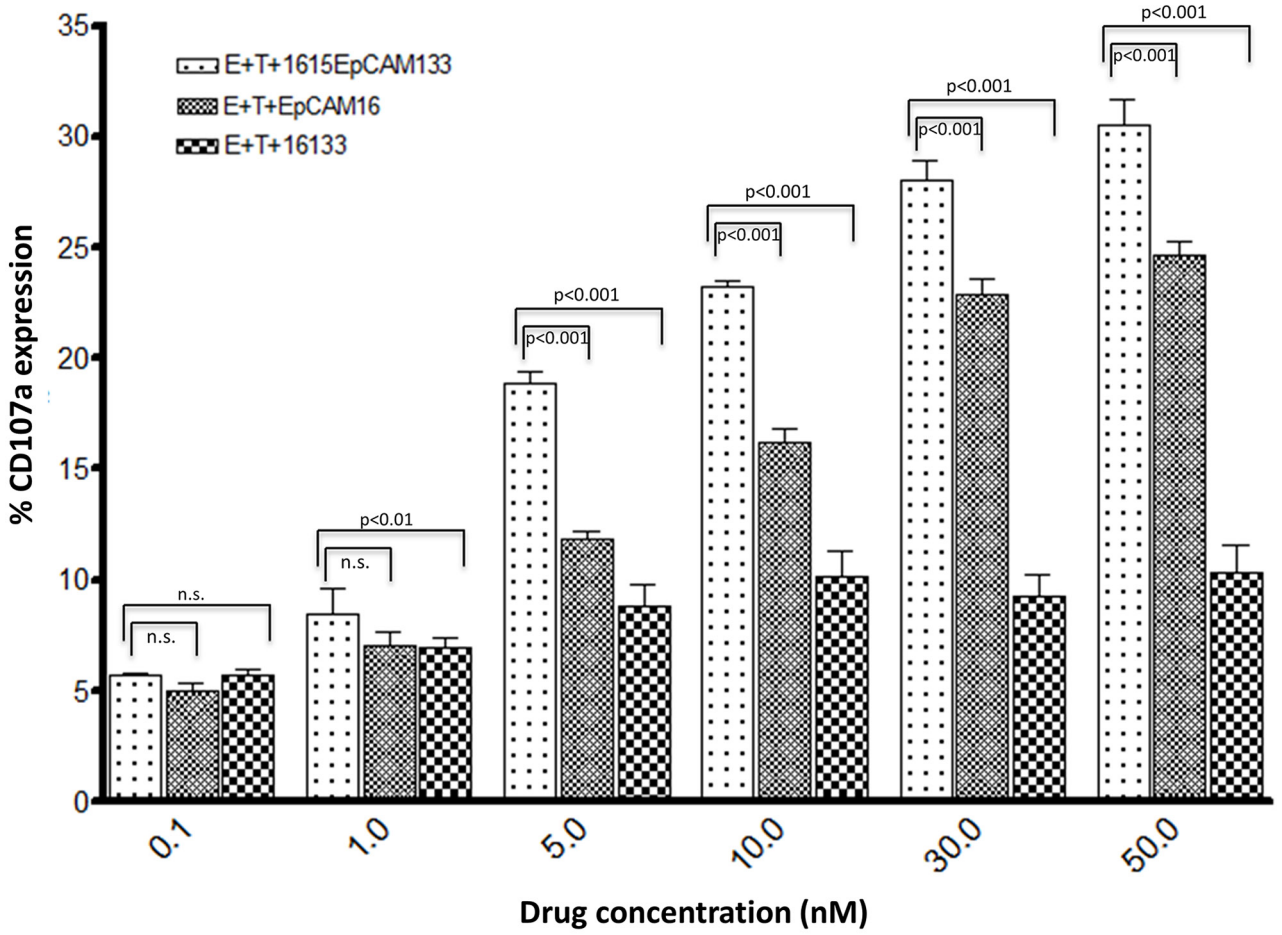
Dose dependent degranulation To quantify dose dependent degranulation a flow cytometry based CD107a assay was performed. CD107a surface expression serves as a marker for NK cell degranulation. PBMC were cultured with cells and 1615EpCAM133 TetraKE, EpCAM16 BiKE or 16133 BiKE at the labeled concentrations (n=3). P-values were estimated with one-way-ANOVA values are presented with standard deviation.

In order to study dose dependent NK cell degranulation, CD107a surface expression was evaluated as it represents a degranulation marker for NK cells and allows for evaluation of degranulation on a per cell basis. PBMCs were cultured with 1615EpCAM133, EpCAM16 and 16133 in increasing concentrations (0.1, 1, 5, 10, 30, 50 nM). At 0.1 nM concentration, no differences in activity were seen. At higher concentrations, the TetraKE showed significantly superior degranulation induction compared to EpCAM16 and 16133. (Figure 4).

### Specificity in degranulation and IFN-γ production

**Figure 5:**
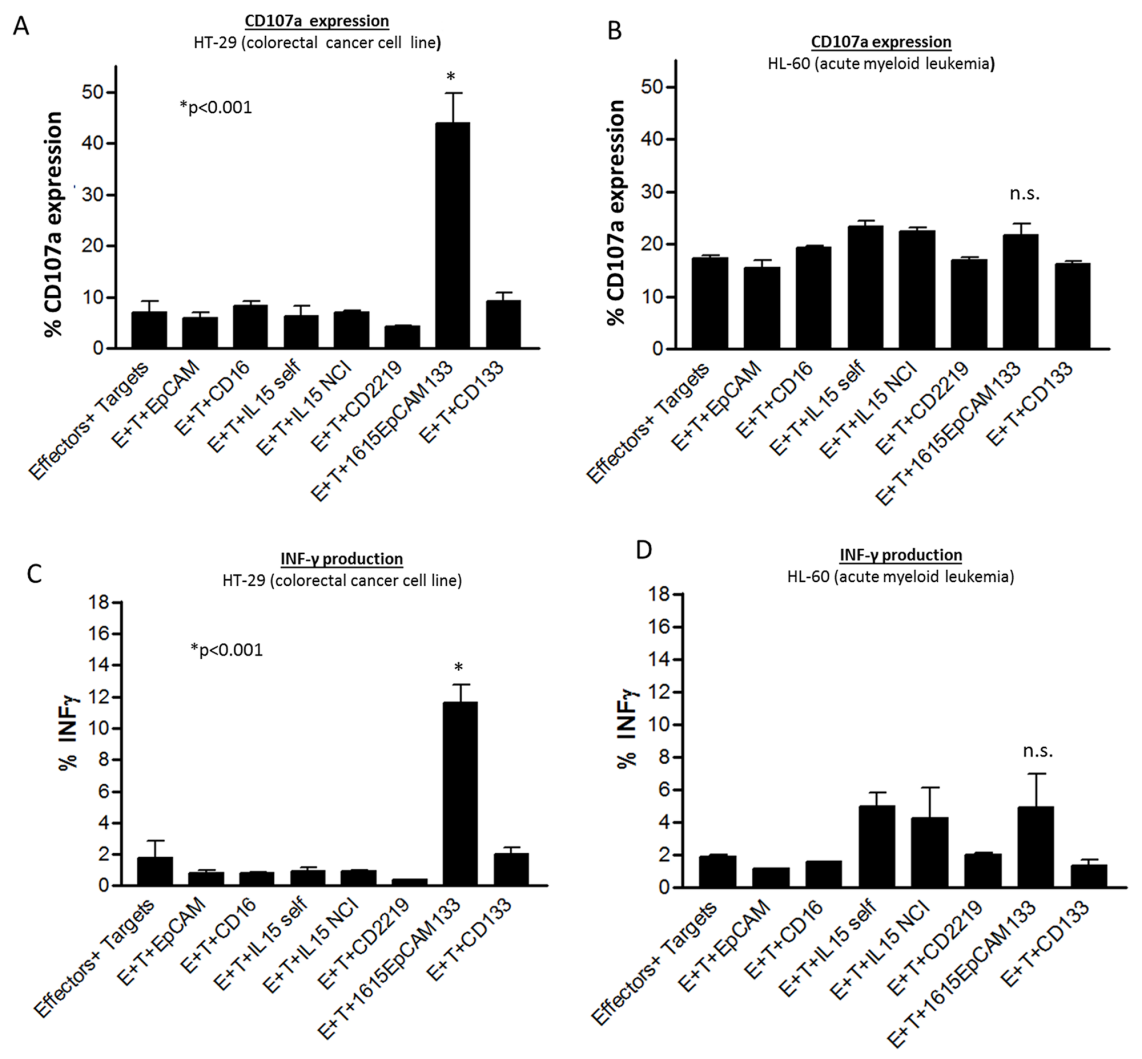
Specificity of degranulation and IFN-γ production **A.** Pooled NK cell CD107a expression data on PBMCs incubated with HT-29 targets or **B.** HL-60 targets with noted drugs (30nM)/stimuli. **C.** Pooled NK cell IFN-γ expression data on PBMC incubated with HT-29 targets or **D.** HL-60 targets with noted drugs/stimuli (n=3). P-values were estimated with one-way-ANOVA values are presented with standard deviation.

For assessment of specificity in induction of lytic degranulation and IFN-γ production, we evaluated CD107a surface expression and intracellular IFN-γ production by flow cytometry and used NK cells co-cultured with HT-29 targets and negative control HL-60 targets. In the HT-29 group, 1615EpCAM133 showed significantly elevated degranulation with target cell exposure (p<0.001) compared to the following controls: Effectors (E)+Targets (T) alone, E+T+anti-EpCAM scFv, E+T+anti-CD16 scFv, E+T+ IL-15 cloned in our lab, E+T+IL-15 NCI derived, E+T+CD2219, E+T+anti-CD133 scFv (Figure 5A). The HL-60 cell line served as a negative control and showed no significant degranulation activity with all applied drugs tested (Figure 5B). IFN-γ production was significantly enhanced against HT-29 cells when NK cells were exposed to the TetraKE (p<0.001) (Figure 5C). NK cells exposed to HL-60 targets showed minimal induction of IFN-γ with monomeric IL-15 or TetraKE, mediated likely by IL-15 signaling, (Figure 5D).

### Degranulation and IFN-γ production against various cell lines

**Figure 6:**
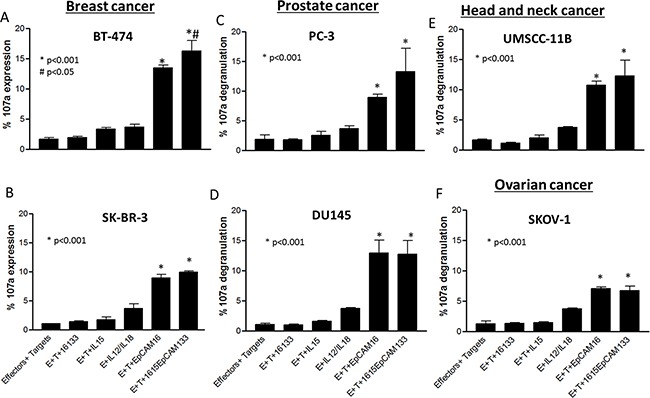
Lytic degranulation in different cancer cell lines. PBMCs were incubated with **A.** BT-474, **B.** SK-BR-3, **C.** PC-3, **D.** DU145, **E.** UMSCC-11B, and **F.** SKOV-1 cell lines and noted drugs (30nM) and CD107a expression was measured on NK cells by flow cytometry. Graphs represent pooled data (n=3). P-values were estimated with one-way-ANOVA values are presented with standard deviation.

TetraKE induces enhanced functionality against a broad range of cancer cell lines. To evaluate drug performance in EpCAM positive cancer entities, various cell lines comprising breast (BT-474 (Figure 6A), SK-BR-3 (Figure 6B)), prostate (PC-3 (Figure 6C), DU145 (Figure 6D)), Head and Neck (UMSCC-11B (Figure 6E)) and ovarian carcinoma (SKOV-1 (Figure 6F)) were analyzed. These respective cancer cell lines were co-cultured with PBMCs and 1615EpCAM133, NCI derived IL-15, 16133 or EpCAM16. With all cell lines, the TetraKE induced variable but higher NK cell CD107a expression compared to controls (p<0.001) and with the breast cancer cell line BT-474, 1615EpCAM133 was significantly superior compared to EpCAM16 BiKE (p<0.05).

**Figure 7:**
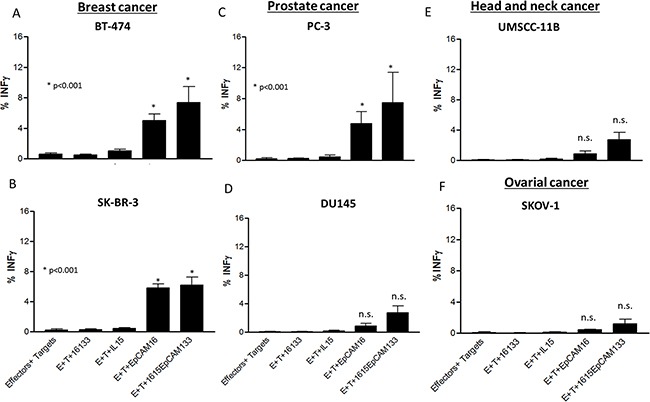
IFN-γ production in different cancer cell lines. PBMCs were incubated with **A.** BT-474, **B.** SK-BR-3, **C.** PC-3, **D.** DU145, **E.** UMSCC-11B, and **F.** SKOV-1 cell lines and noted drugs (30nM) and IFN-γ expression was measured on NK cells by flow cytometry. Graphs represent pooled data. P-values were estimated with one-way-ANOVA values are presented with standard deviation.

The same effectors and target cells were evaluated for IFN-γ production. All breast cancer subtypes as well as PC-3 prostate cancer cell line (Figure 7A, 7B, 7C) induced significantly elevated NK cell IFN-γ production (p<0.05) when exposed to the TetraKE and EpCAM16 BiKE. All other cell lines shown in Figure 7 (DU145 (D), UMSCC-11B (E) and SKOV-1 (F)) showed no significantly enhanced IFN-γ production with TetraKE or of BiKE compared to controls.

### Cytokine production

**Figure 8:**
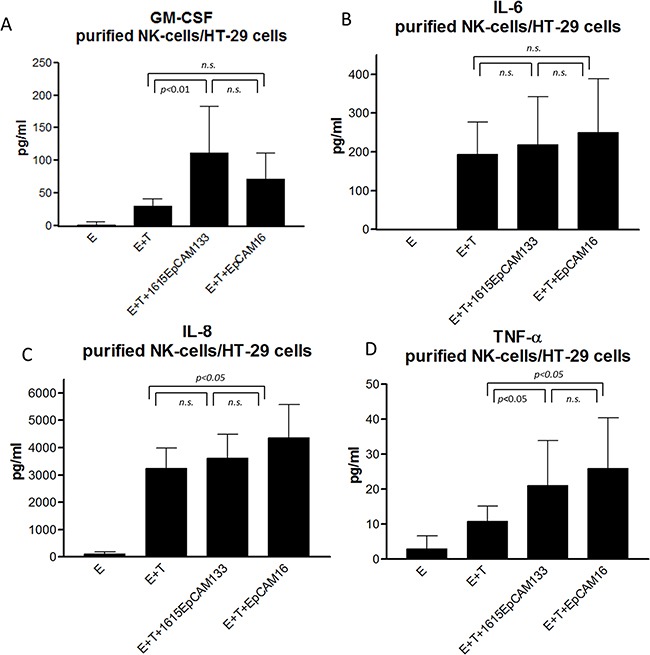
Cytokine profile Purified NK cells from healthy donors [E] were cocultured with HT-29 colon carcinoma cell line [T] and 1615EpCAM133 TetraKE, EpCAM16 BiKE, no drug and NK cells alone. Hallmark cytokines for inflammation were tested in supernatants via Luminex. **A.** GM-CSF, **B.** IL-6, **C.** IL-8, and **D.** TNF-α production is displayed pg/ml in the pooled data graphs (n=6). P-values were estimated with one-way-ANOVA values are presented with standard deviation.

In order to evaluate a broader cytokine profile, purified NK cells were co-cultured with HT-29 colon carcinoma cell line. After 24 hours of incubation, supernatant was analyzed for hallmark cytokines of inflammation. A significant difference in GM-CSF production between 1615EpCAM133 to no drug (p<0.01) but no difference between TetraKE and EpCAM16 BiKE was seen (Figure 8A). Concerning IL-6 production, no difference between TetraKE and BiKE was noted (Figure 8B). IL-8 production showed no difference between TetraKE and BiKE (Figure 8C). TNF-α evaluation showed no difference between TetraKE and BiKE but we saw a significant difference between both drugs compared to E+T alone (p<0.05) (Figure 8D).

## DISCUSSION

The original contribution of this work is a self-sustaining TetraKE comprising an anti-CD16 scFv for NK cell engagement, anti-EpCAM scFv for carcinoma recognition, anti-CD133 scFv for CSC recognition, and an IL-15 cross-linker to sustain the NK cell response. Our TetraKE is capable of inducing dual antigen specific ADCC and specific delivery of an IL-15 signal to the NK cell-target synapse. This leads to improved activity, induction of proliferation, and prolongation of survival of NK cell effectors. These properties were not seen in an anti-EpCAM BiKE [6] and an anti-CD133 BiKE [4] previously reported by our group. Our group has developed IL-15 TriKEs targeting singular cancer-associated markers [26, 27]. However, this is the first of its kind IL-15 driven TetraKE targeting two cancer markers simultaneously, one of them an established marker on cancer stem cells, CD133 [28].

The central core of NK cell mediated ADCC by an engineered bispecific antibody is targeting the Fcγ receptor III (CD16) with one ligand and a cancer cell target with the other ligand. This forms an immune synapse resulting in cytotoxic degranulation. This construction shows efficacy in targeting hematologic malignancies [7, 29] as well as solid tumors [4]. Regarding hematologic cancers, a phase I clinical trial with a bispecific monoclonal antibody simultaneously targeting CD16 and the Hodgkin and Reed-Sternberg cell-associated CD30 antigen (HRS-3/A9) showed one complete and one partial remission (CR/PR) out of 9 patients [30]. In a second randomized pilot trial, the same drug showed one CR and three PR out of 16 patients [31]. Gleason et al. evaluated a BiKE and a TriKE scFv construct targeting CD16/CD19 and CD16/CD19/CD22 expressed on B-cell malignancies and showed stability of the constructs in human serum and selective killing of a Burkitt-lymphoma cell line [5]. Kugler et al. created a dual targeting scFv triplebody specific for Interleukin-3 receptor α chain (CD123), CD33 and CD16 showing responses against acute myeloid leukemia (AML)-cell lines [8]. Focusing on solid tumor markers, 2B1, a bispecific murine monoclonal antibody with bispecificity for ErbB2 and CD16 was tested in a phase I clinical trial and showed responses in breast cancer patients [32]. A BiKE produced for targeting CD16 and EpCAM receptors killed various EpCAM bearing cell lines [6]. An anti-CD133 BiKE killed CD133^+^ colorectal cells [4]. Although highly effective, none of these BiKE constructions were as capable of a self-sustained NK expansion as our IL-15 TetraKEs.

IL-15 is the homeostatic regulator of proliferation, differentiation, activation and survival of NK cells. IL-2 has also been used to improve NK cell performance against various solid tumors [33, 34]. However cytokine toxicity led to dose reduction and outcome showed minor IL-2 related advantages. A study from Munger et al. used a murine model to show that major side effects of IL-2 such as capillary leak syndrome were not present after treatment with IL-15 [35]. Furthermore IL-15 regulates and initiates anti-apoptotic signals in NK cell effectors and thereby performs proliferation and survival properties [36]. This has been shown to lead to an increase of NK cells up to 3-fold and measurable response in patients with solid tumors [9]. IL-15 effects represent beneficial characteristics in cancer treatment, which can be specifically delivered to the effector cells with our TetraKE.

One potential advantage of delivering IL-15 directly to the tumor with TetraKE is that systemic exposure might be avoided reducing toxicity. IL-15 can induce proinflammatory cytokines such as IL-6, IL-8, TNF-α, IL-1β and GM-CSF [37]. This was shown in patient studies with systemic IL-15 treatment. After IL-15 administration, acute toxicities such as hemodynamic instability, fever and chills were reported [9]. Furthermore systemic administration of IL-15 administration was revealed to have myelosuppressive properties like induction of neutropenia and thrombocytopenia [9]. To address the potential of a critical cytokine release observed in several immune engagers especially with the IL-15 moiety, IFN-γ, a critical cytokine associated with toxicity was studied and evaluated after TetraKE exposure. Results were encouraging, showing only a moderate IFN-γ induction compared to the control with supraphysiologic levels induced by IL-12/IL-18 (data not shown). Similar results with a moderate cytokine secretion were also seen by performing a cytokine profile measuring GM-CSF and TNF-α, which showed enhanced cytokine production with the TetraKE compared to no treatment but not significantly more compared to the EpCAM16 BiKE without the IL-15 moiety. It remains unclear if further IL-15 related effects such as promotion of proliferation in patients with lymphoproliferative disease [38] and impact in angiogenesis, invasion, and hyperplasia [39] can be induced by the IL-15 moiety. Based on our observations we believe that the specific delivery to the immunologic synapse might protect from systemic and unfavorable IL-15 effects, which will require clinical testing.

According to the “stem cell model” a small group of stem cells undergo an asymmetric cell division to either equal stem cells or to more differentiated progenitor cells which in turn can provide more differentiated cells inside the tumor mass. This CSC population expresses CD133, is chemotherapy- and radiotherapy resistant, and has tumor initiating and self-renewal abilities [40]. The critical role of CSC has been reported in solid tumors such as breast, colon, prostate, liver, pancreatic, lung cancer and head and neck squamous cell carcinoma (HNSCC) [41]. Cancer can be inhibited by eliminating CSC. A CD133 targeting toxin and a CD133 BiKE which were constructed with the same CD133 binding site as the TetraKE showed good response rates in CD133^+^ bearing ovarian, gastrointestinal and breast cancer [23, 42–44] even when the CD133^+^ population was <10% CD133^+^ cells. [43]. CD133KDEL, a targeted toxin showed specific and dose dependent inhibition in human HNSCC cells in vivo by our group [22]. Importantly, these studies showed the ability to eliminate tumor initiating cells (e.g. CSC) in a tumor xenograft model. For this reason, this very same anti-CD133 scFv was used in the construction of 1615EpCAM133 in this paper. This dual targeting should provide an enhanced strategy to effectively target the tumor.

EpCAM is a frequently expressed marker on carcinoma cells [45] that has impact on patient prognosis [45]. Thus, it was chosen for our studies. Other immune engagers have been developed that successfully target EpCAM and have been tested clinically with some success validating this target choice. Catumaxomab, is a BiTE that targets CD3 on T cells and EpCAM. In a randomized clinical trial, there was a significant decrease in the need for drainage [46] in patients suffering from EpCAM^+^ tumor associated ascites. In some reports, shrinkage of distant metastasis after therapeutic intraperitoneal administration occurred [47]. Another bispecific single-chain antibody construct was created targeting the same epitopes (MT110) as Catumaxumab. Successful eradication of pancreatic cancer derived CSC in vitro and in a murine mouse model was observed [48]. Furthermore Brischwein et al. tested this construct for efficacy on nine EpCAM bearing cell lines comprising breast, colon and gastric cancer. All cell lines were susceptible to redirected lysis [49]. A deimmunized bispecific targeted toxin (Pseudomonas enterotoxin) against EpCAM and CD133 showed efficacy against UMSCC-11B head and neck carcinoma in a xenograft murine model [23].

EpCAM is commonly expressed on all epithelial tissues, albeit in lower copy numbers. In order to evaluate the safety profile, Catumaxumab was tested for toxicity in a moiety of murine animal models. Side effects were described with pyrexia, hepatotoxicity, nausea and bone marrow suppression [50] and might be related to T cell activation and systemic cytokine release. By harnessing NK cell effectors to eliminate tumor cells and presenting IL-15 locally in the immune synapse, we expect to achieve a better safety profile [4, 6]. However*, in vivo* experiments will be required to test this prospectively in a murine model since human IL-15 cross-reacts with mice.

In conclusion, our work presents a potent TetraKE with improved dual antigen ADCC. By targeting CD133 and EpCAM simultaneously, our TetraKE addresses the polygenetic nature of most cancers and enhances probability to attack cancer at its basal root, the CSC. Importantly, the addition of IL-15 to the platform induces effector cell proliferation/activation and prolongs effector cell survival. With these improvements, we believe that our drug represents a new generation of NK cell engagers with improved efficacy in anti-cancer treatment.

## MATERIALS AND METHODS

### Construction of 1615EpCAM133

Synthesis and assembly of hybrid genes encoding 1615EpCAM133 was accomplished using DNA shuffling and DNA ligation techniques. The fully assembled gene (from the 5′ end to 3′ end) consisted of an NcoI restriction site, an ATG initiation codon, the V_H_ and V_L_ regions of human CD16 (NM3E2) derived from a phage display library produced by McCall et al. [51], a 20 amino acid (aa) segment (PSGQAGAAASESLFSNHAY), modified (N72D) IL-15 [52], a seven aa segment (EASGGPE), the humanized anti-EPCAM scFv from the antibody MOC-31, a 15 aa mutated human IgG1 hinge region (EPKSSDKTHTSPPSP), the anti-CD133 scFv from clone 7 [53], and finally a NotI restriction site. The resultant 2715 bp NcoI/NotI fragment gene was spliced into the pET28c expression vector under control of an isopropyl-β-D-thiogalactopyranoside (IPTG) (FischerBiotech, Fair Lawn, NJ, USA) inducible T7 promoter. DNA sequencing analysis (Biomedical Genomics Center, University of Minnesota, MN, USA) was used to verify that the gene was correct in sequence and had been cloned in frame. Genes for monospecific anti-CD16 scFv, anti-EpCAM scFv, and anti-CD133 scFv were created in the same manner. Molecular weight was calculated with “SerialCloner software 2-6-1” according to known amino acid structure gained from the cloned sequence and was 95,900 Da.

### Inclusion body isolation

Bacterial protein expression was performed with *Escherichia coli* strain BL21 (DE3) (Novagen, Madison WI, USA) by plasmid transformation. Bacteria were cultured in 1000 ml Luria broth containing 30 mg/ml kanamycin. Gene expression was induced when culture media reached an OD600 of 0.65 with the addition of 1 mM IPTG. Three hours after induction bacteria were harvested (from 4 liters cultured media we isolated 38 g bacterial pellet). After harvesting the pellet the cell paste was suspended in 375 ml Lysis buffer solution (50 mM tris, 50 mM NaCl, and 5 mM EDTA pH 8.0), sonicated and centrifuged. For pellets extraction they were washed four times in detergent buffer (1% sodium deoxycholate, 15% Triton X-100, 10% glycerin, 50 mmol/L Tris, 50 mmol/L NaCl, 5 mmol/L EDTA) (pH 8.0) and four in Lysis buffer.

### Refolding and purification

Refolding and purification was recently described [4]. In order to refold proteins from inclusion bodies (IB), the 5 gram pellet of partially purified inclusion bodies was dissolved into Solubilization buffer (100mM Tris, 2.5% SLS, pH 10.3) and stirred for 5 hours at room temperature. CuSO_4_ was added to the supernatant to oxidize the protein. 6M urea was added to the oxidized inclusion bodies and stirred at room temperature for one hour. 15% AG 1-x8 Resin was added and incubated by stirring lightly for 20 minutes at room temperature to remove SLS. The AG 1-x8 Resin was removed by filtering through 0.45 micron filter. 7 M Guanidine-HCl was added to the filtered samples and stirred for 2 hours. Sample was diluted into cold refolding buffer (50mM Tris, 0.5 M L-Arginine, 1M Urea, 20% Glycerin, 50mM NaCl, 5mM EDTA, pH 8.0) and incubated at 4°C for 72 hours. The refolded 1615EpCAM133 was dialyzed first against 20mM Tris-HCl + 1M Urea then 20mM Tris-HCl without Urea two times. The 1615EpCAM133 was purified over FFQ Sepharose anion exchange and Superdex 200 sizing columns. Using SDS-PAGE we evaluated purity. The fusion proteins were stained with Simply Blue life Stain (Invitrogen, Carlsbad, CA, USA) and size was confirmed with molecular weight standards.

### Cell lines

Cancer cell lines Caco-2^a^, HT-29^b^ (human colorectal carcinoma cell lines), SK-BR-3^c^, BT-474^b^ (breast cancer cell lines), PC-3^b^, DU-145^a^ (prostate cancer cell lines), UMSCC-11B^c^ (head and neck cancer cell line) and SKOV-1^d^ (ovarian cancer cell line) were obtained from American Type Culture Collection (ATCC, Rockville, MD, USA) and grown as a monolayer in tissue culture flasks as published [54, 55]. HL-60^b^ (promyelocytic leukemia cell line) was also obtained from ATCC and grown in suspension culture [56]. Media for cell culture was used as appropriate: ^a^EMEM with 20%, ^b^RPMI, ^c^MEM, ^d^DMEM with 10% fetal bovine serum. Additionally to the preceding supplements, BT-474 media contained 10 μg/mL insulin. All medias were supplemented with 2 mmol/L L-glutamine, 100 μg/mL streptomycin and 100 units/mL penicillin. Cell cultures were incubated in a 37°C atmosphere containing 5% CO_2_. Adherent cells were passaged by using trypsin-EDTA for detachment when 80-90% confluent. Cells were counted with a standard hemocytometer and used after showing viability of >95% in trypan blue staining.

### NK cell isolation and purification

To isolate peripheral blood mononuclear cells (PBMC), a histopaque gradient (Sigma-Aldrich, St. Louis, MO, USA) and SepMate^TM^ tubes (Stemcell technologies, Vancouver, Canada) were used with adult blood (Memorial Blood Center, Minneapolis, MN, USA) of healthy volunteers. Samples were obtained after informed consent and in accordance with the University of Minnesota human subjects Institutional Review Board and the Declaration of Helsinki. In case of NK cell purification, standard magnetic bead enrichment was used according to the manufacturer's protocol (Stemcell Technologies, Vancouver, BC, Canada).

### Flow cytometry based binding and blocking assay

For the binding assay 4×10^5^ of Caco-2 or HT-29 cells were used. After washing, cancer cells were incubated in 4°C with 200nM Fluorescein isothiocyate (FITC)-labeled 1615EpCAM133 TetraKE for 30 minutes. For the blocking 1000 nM of either anti-EpCAM scFv, anti-CD22-CD19 scFv (anti-CD22 scFv linked to anti-CD19 scFv), anti-CD133 scFv or CD133EpCAM (anti-CD133 scFv linked to anti-EpCAM scFv) was used. After washing procedures, staining intensity was evaluated with the LSRII flow cytometer (BD Biosciences, San Jose, CA, USA).

### Flow-cytometry based CD107a degranulation assay

Procedures were previously reported [5]. Isolated PBMCs were washed and then incubated overnight (37°C, 5% CO_2_) in RPMI 1640 supplemented with 10% fetal bovine serum. Cells were washed and treated with the respective concentrations of 1615EpCAM133, EpCAM16, anti-EpCAM scFv, IL-15 (NCI derived), anti-CD133 scFv and incubated for 10 minutes at 37°C with 5% CO_2_. FITC-conjugated anti-human CD107a monoclonal antibody (mAb) (LAMP-1) (BD biosciences, New Jersey, CA, USA), was added and further incubated for 1 hour. GolgiStop (1:1500) (BD Biosciences, San Jose, CA, USA) and GolgiPlug (1:1000) (BD Biosciences, San Jose, CA, USA) were added and cells were further incubated for 3 hours. Cells were washed and stained with the following mAb from BioLegend, San Diego, CA, USA: PE/Cy7-conjugated anti-CD56; APC/Cy 7-conjugated anti-CD16; PE-CF594-conjugated anti-CD3. After incubation for 30 minutes, cells were fixed with 2% paraformaldehyde. For intracellular staining, Pacific Blue-conjugated anti-human IFN-γ (BioLegend, San Diego, CA, USA) was used after permeabilization with permeabilization buffer (BD Biosciences, San Jose, CA, USA). After incubation for 15 minutes cells were washed and evaluated by FACS analysis using a LSRII flow cytometer (BD Biosciences, San Jose, CA, USA).

### Chromium-51 release cytotoxicity assay

Caco-2 and HT-29 target cells were labeled for 1 hour with 1μCi of ^51^Cr per 1×10^5^ target cells at 37°C, 5% CO_2_. Washing procedures were performed to remove excess ^51^Cr. The labeled target cells were added to the wells of 96-well round-bottom plates (5×10^3^ cells). Effectors were treated with 1615EpCAM133 TetraKE or negative controls and then added to the plates. Effector: Target (E:T) ratio ranged between 20:1 and 2.2:1. The amount of ^51^Cr released, which corresponds to target cell death, was measured by a gamma scintillation counter, and the percent target cell lysis was calculated as follows: [(experimental lysis - spontaneous lysis)/(maximal lysis - spontaneous lysis)] x 100. To determine maximal lysis, ^51^Cr-labeled target cells were treated with 3% Triton X for 4 hours.

### Luminex

In order to analyze chemokines and cytokines, we used purified NK cells from 6 healthy volunteers and coincubated the cells in 96 well plates for 24 hours with HT-29 cells at a 2:1 E:T ratio. In addition the respective drugs were added in a concentration of 50nM. Cells were stored at 37°C, 5% CO_2_ and incubated for 24 hours. Then supernatants were collected and stored at −8o°C until analysis. GM-CSF, IL-6, IL-8 and TNF-α (R&D Systems, Minneapolis, MN, USA) levels were determined using the Luminex system (MAGPIX, Luminex, Austin, TX, USA). The values, presented in pg/ml, were extrapolated from standard curves of the recombinant human proteins using Xponent 4.2 software (Luminex, Austin, TX, USA).

### Proliferation assay

In order to determine proliferation, PBMC of purified NK cells from healthy volunteers were obtained and labeled with CellTrace Violet Cell Proliferation Dye (Invitrogen, Carlsbad, CA, USA) as described in the manufacturer's protocol. Then cells were cultured with 50nM of the respective drug. After 7 days cells were harvested, stained for viability with Live/Dead stain (Invitrogen, Carlsbad, CA, USA), and for surface markers anti-CD56 PE/Cy7 (Biolegend, San Diego, CA, USA) and anti-CD3 PE-CF594 (BD Biosciences, Franklin Lakes, NJ, USA) to gate on the viable CD3^−^CD56^+^ NK cell population. Data analysis was carried out on FlowJo software version 7.6.5. (Flowjo enterprise LCC, Ashland, OR, USA).

### Statistical analysis

Data are presented as mean +/− standard deviation. Differences between groups were analyzed by Student's t test or one-way-Anova. Analysis and presentation of data was done with Graphpad prism 5 (GraphPad Software, Inc., La Jolla, CA, USA).
